# Endovascular treatment of ureteroarterial fistula using a covered stent, evaluated by intravascular ultrasound: a case report

**DOI:** 10.1186/s42155-019-0060-6

**Published:** 2019-05-16

**Authors:** Kazunori Horie, Toshiya Fujiwara, Kiyofumi Satoyoshi, Masato Munehisa, Naoto Inoue

**Affiliations:** 1 Division of Cardiovascular Medicine, Sendai Kousei Hospital, 4-15 Hirose-cho, Aoba-ku, Sendai, Miyagi 980-0873 Japan; 2 Department of Cardiovascular Medicine, Akita General Hospital, Akita, Japan; 3 Department of Urology, Akita General Hospital, Akita, Japan

**Keywords:** Ureteroarterial fistula, Endovascular therapy, Covered stent-graft, Coil embolization, Intravascular ultrasound

## Abstract

**Background:**

Ureteroarterial fistula is a rare life-threatening complication of indwelling ureteral stents. The mechanism has not yet been fully evaluated using intravascular imaging.

**Case presentation:**

An-84-year-old female was referred to our unit because of large volume pulsatile bleeding from the left ureter during routine stent exchange in the urology department. The hematuria was initially managed by rapidly exchanging for a new stent; however, the patient went into hypovolemic shock due to acute blood loss. The patient underwent implantation of the bilateral ureteral stents due to urinary retention caused by retroperitoneal fibrosis 2 years ago. To prevent ureteral infection, occlusion of the stents and stone formation, the stents were exchanged every 6 months. Computed tomography revealed contact between the left ureter and the common iliac artery. Therefore, ureteroarterial fistula was suspected and endovascular therapy was performed. Although angiography did not show definite blood flow into the ureter, a soft guidewire was advanced from the subintima of the external iliac artery to the left ureter. The diagnosis of ureteroarterial fistula was confirmed. Intravascular ultrasound identified the stent in the ureter and its connection to the subintima of the external iliac artery. The ureter did not contact directly to the inner lumen of the iliac arteries according to the ultrasound findings; therefore, we considered that the risk of stent-graft infection might not be high. After coil embolization of the ipsilateral internal iliac artery, a covered stent was implanted in the external iliac artery to seal the subintimal entry. The patient had no further episodes of any gross hematuria on dual anti-platelet therapy, when the ureteral stent was exchanged three time during 1 year after the endovascular therapy.

**Conclusions:**

We demonstrated a case of ureteroarterial fistula, in which intravascular ultrasound allowed to visualize the communication between the ureter and the subintimal lumen in the external iliac artery.

**Electronic supplementary material:**

The online version of this article (10.1186/s42155-019-0060-6) contains supplementary material, which is available to authorized users.

## Background

Ureteroarterial fistula (UAF) is an uncommon condition first described in 1908 by Moschcowitz (Moschcowitz 1908). It occurs as a result of a fistulous communication between a ureter and an aorta or iliac artery. UAF is classified into primary (15%) and secondary (85%) types based the cause (Pillai et al. 2015). Secondary causes result from pelvic cancer (70.3%) and prior interventions including surgery (69.5%) combined with radiation (48.3%), and the most common risk factor was presence of a chronic indwelling ureteral stent (73.7%) (Das et al. 2016). The mechanical fixation of the ureter appears to lead to inflammation and fibrosis in the adjacent artery during pulsation; however, the mechanism has not yet been fully evaluated using intravascular imaging. We report a case of UAF caused by a ureteral stent placement in which intravascular ultrasound (IVUS) was used to evaluate the communication through the subintimal lumen of the external iliac artery (EIA).

## Case presentation

**Fig. 1:**
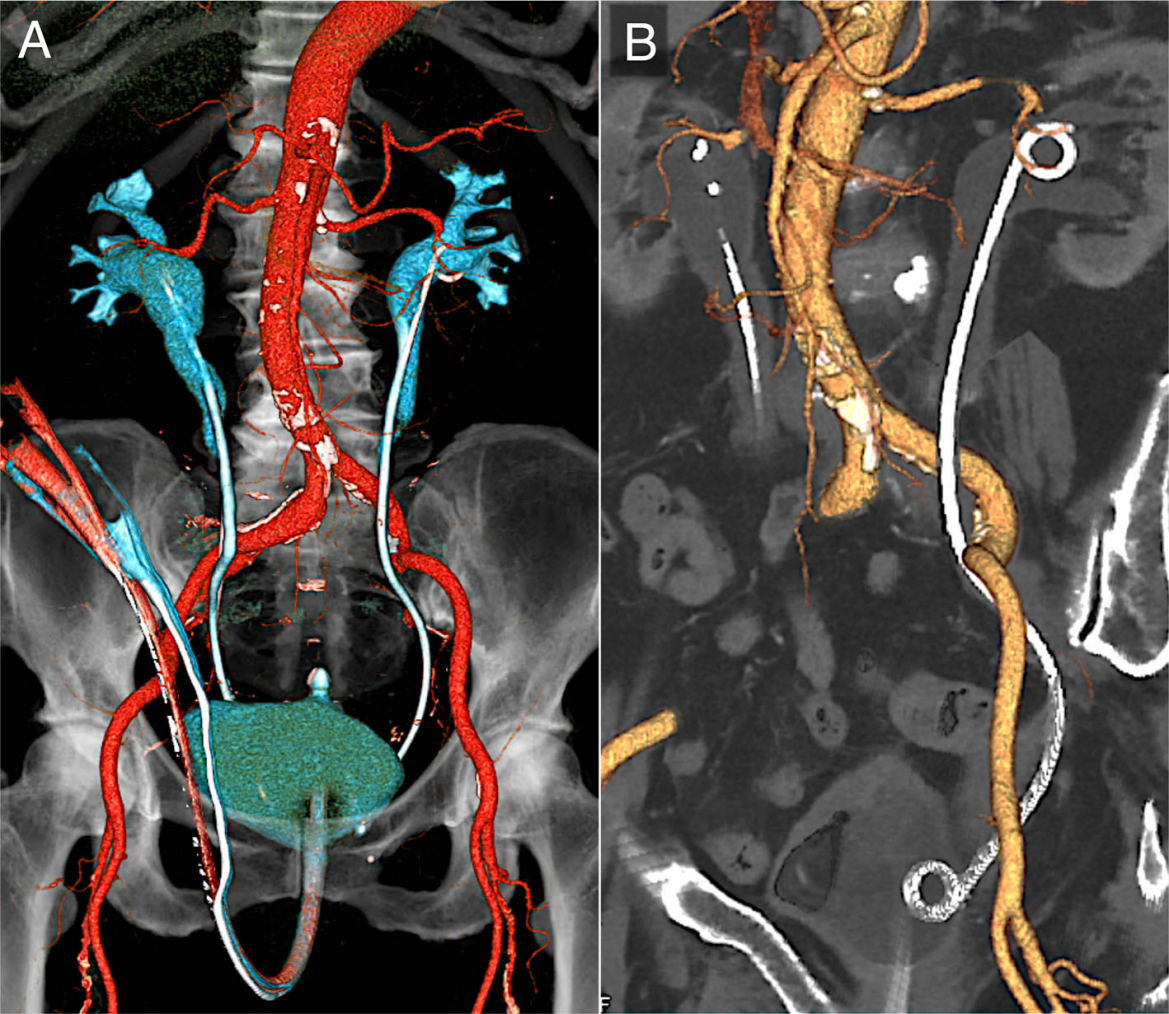
Computed tomography after the hemostasis of hematuria. **a** Computed tomography (CT) showed the crossing of the left ureter with a stent over the distal common iliac and internal iliac arteries. **b** CT imaging in the angulated 30-degree left anterior oblique position

**Fig. 2:**
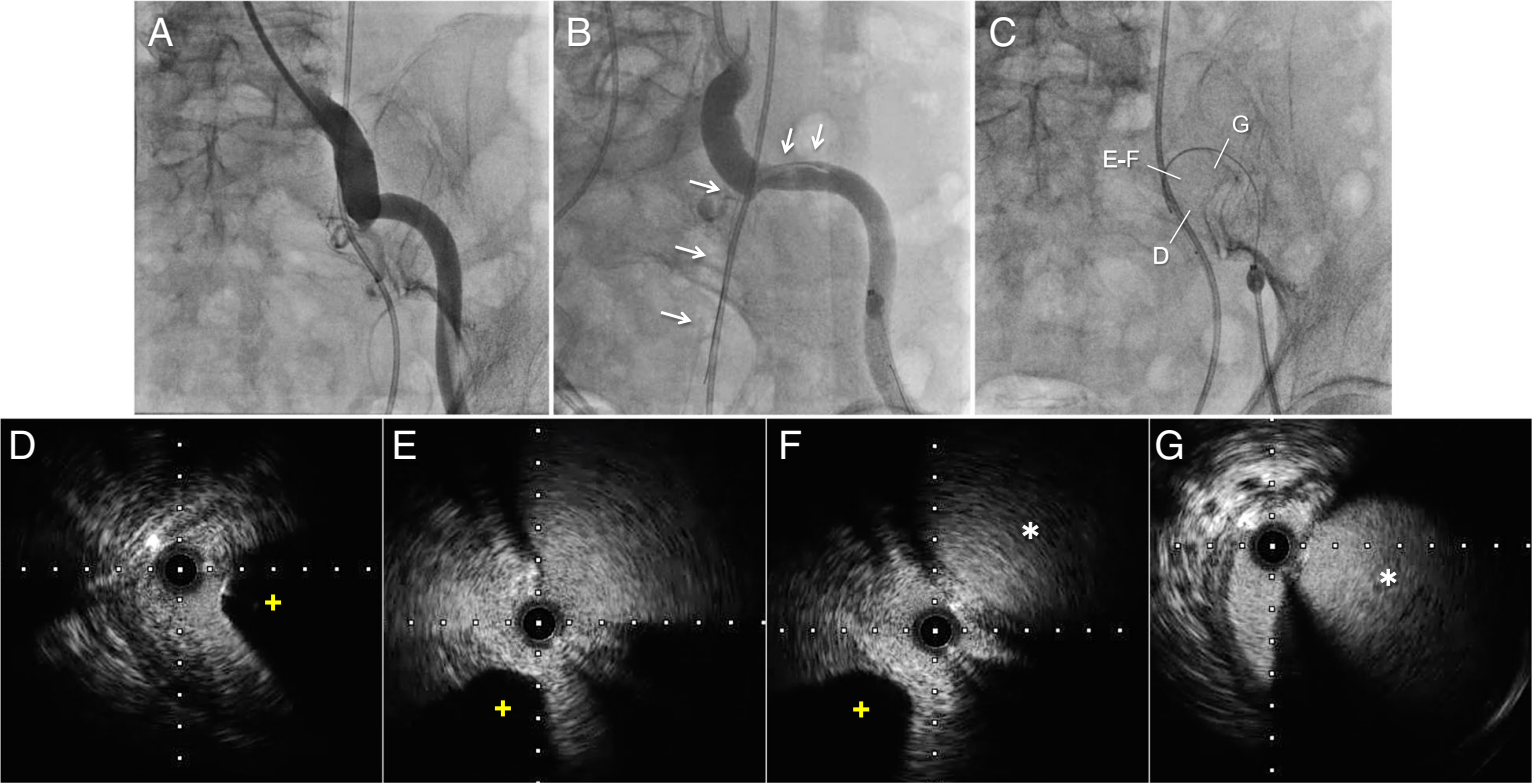
The endovascular procedure and the findings of intravascular ultrasound. **a**, Baseline angiography did not show the blood flow into the left ureter from the left iliac arteries. **b** and **c** A 0.014-in. guidewire was advanced into the left ureter along the stent from the subintimal lumen of the external iliac artery (*white arrows*). The anatomical findings were evaluated by intravascular ultrasound (IVUS). **d** IVUS detected the stent with an acoustic shadow (*yellow plus signs*) in the left ureter. **e**–**g** IVUS detected the communication between the ureter and subintimal lumen of the EIA (*white asterisks denote true lumen of the left external iliac artery*)

**Fig. 3:**
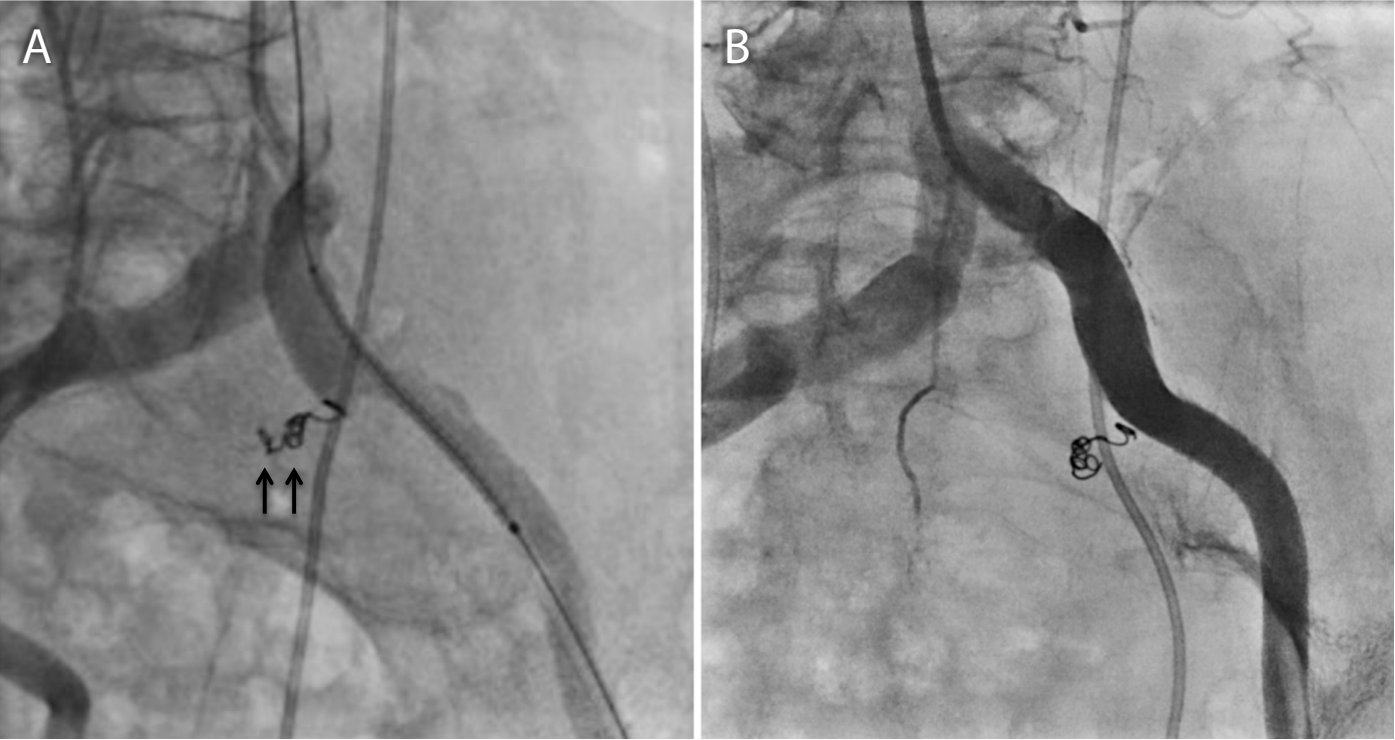
Implantation of the covered stent and coil embolization. **a** A Viabahn stent graft of 9.0 × 50 mm was implanted to cover from the crossing of the left ureter and iliac artery to the distal entry of the subintimal lumen (*black arrows denote coils in the left internal iliac artery*). **b** The final angiography revealed complete closure of the subintimal lumen and the internal iliac artery

An-84-year-old female was referred to our unit after the Urologist encountered large volume pulsatile bleeding from the left ureter during routine stent exchange. The hematuria was initially managed by rapidly exchanging for a new stent; however, the patient went into hypovolemic shock due to acute blood loss. The patient had a history of urinary retention due to retroperitoneal fibrosis caused by immunoglobulin G4-related disease 2 years ago. Ureteral stents were placed in the patient’s bilateral ureters. To prevent ureteral infection, occlusion of the stents and stone formation, the stents were exchanged every 6 months. After the hematuria, computed tomography (CT) scan did not show the injury of the left kidney and ureter; however, revealed contact between the ureter and common iliac artery (CIA) (Fig. 1a-b, axial imaging in Additional file 1: Movie S1 and sagittal in Additional file 2: Movie S2). Therefore, UAF was suspected. In order to facilitate the need for ongoing exchanges of the ureteral stent in the future, endovascular therapy (EVT) was performed. A 6.0-Fr sheath was placed via the left common femoral artery and a 4.5-Fr guiding sheath with a length of 120 cm was inserted via the left radial artery. Baseline angiography did not show blood flow into the ureter from iliac arteries (Fig. 2a and Additional file 3: Movie S3) (Das et al. 2016). The CIA and EIA were too large for OPTICROSS IVUS™ compatible with 0.014-wires (Boston Scientific, MA, US) to identify the connection of the UAF. Next, we considered that angiography via a micro-catheter could evaluate the connection between the internal iliac artery (IIA) and the left ureter. We attempted to advance a 0.014-in. Gladius guidewire™ (Asahi Intecc, Aichi, Japan) to the IIA; however, the wire proceeded from the EIA to the left ureter unintentionally (Fig. 2b and c). The diagnosis of UAF was confirmed. IVUS allowed to visualize the stent in the ureter and the subintimal lumen of the EIA without evidences of a definite tract and aneurysm in the connection (Fig. 2d-g and Additional file 4: Movie S4). As mechanical stimulation of the ureteral stent could enlarge the subintimal space of the iliac arteries and cause a new UAF to the ipsilateral IIA in the future, coil embolization of the IIA was performed using three Interlocking Detachable Coils™ (two 2.0 mm × 4.0 cm and one 3.0 mm × 6.0 cm; Boston Scientific, MA, US) via the 4.5-Fr guiding sheath. The ureter did not contact directly to the inner lumen of the iliac arteries according to the IVUS findings; therefore, we considered that the risk of stent-graft infection might not be high. Then, after replacing the 6.0-Fr with a 9.0-Fr sheath, a Viabahn covered stent™ of 9.0 × 50 mm (W.L. Gore & Associates, Flagstaff AZ, US) was implanted from the common iliac artery (CIA) to the entry of the subintima in the EIA (Fig. 3a). Because angiography revealed type I endoleak into the EIA, an Assurant balloon-expandable stent™ of 10 × 30 mm (Medtronic, Frauenfeld, Switzerland) was implanted in the proximal portion of the CIA to press the covered stent to the arterial wall. Final angiography showed closure of the IIA, subintimal lumen, and endoleak (Fig. 3b). The left ureteral stent was exchanged immediate after the EVT, and hematuria did not occur. The patient had no major post-operative complications and was discharged from the hospital. The ureteral stents were exchanged three time during 1 year after the EVT, and the patient had no further episodes of any gross hematuria on dual anti-platelet therapy. The left ankle brachial index was within normal limit at 1 year after the EVT.

## Discussion

UAF is a rare complex problem involving multiple organ systems, usually occurring in patients with significant comorbid conditions due to malignancy, irradiation, previous surgical interventions and indwelling ureteral stents (Das et al. 2016; Turo et al. 2018). A previous report demonstrated that a pseudoaneurysm was detected in up to 38% of cases with UAF, and CT could show an enhancing mass near the crossing of the ureter (Van den Bergh et al. 2009). However, the reported diagnostic rates with CT are only 42–50% in cases without aneurysms, because it is often difficult to detect a direct fistulous communication between the artery and ureter via cross-sectional imaging (Van den Bergh et al. 2009). The current review demonstrated that the angiography was the best modality for the diagnosis of UAF; however, the angiography could detect the bleeding in still only 72.4% (Das et al. 2016). Contrast extravasation into the ureter might not occur when a ureteral stent or clots are at the site and tamponade the leak. Although the baseline angiography did not reveal the definite blood flow into the ureter and EIA in our case, the soft wire proceeded into the ureter from the EIA through the subintimal lumen which was visualized by the IVUS. The subintimal lumen of EIA might be made by the stimulation of the ureteral stent, and this was one of the mechanisms of UAF after ureteral stent implantation. To the best of our knowledge, our case report is the first to demonstrate the morphological findings of UAF using intravascular imaging. Although the diagnosis was made by unintentional wire crossing of UAF in the present case, it would be preferable that imaging examinations confirmed the evidence of UAF before the interventions. Vision PV-.035 IVUS™ compatible with 0.035-in. wires (Philips/Volcano, Amsterdam, the Netherlands) might be effective to observe the whole walls of iliac arteries because the penetration depth is superior to that with 0.014-in. wires.

Treatment of UAF includes excision of the involved arterial segment with extra-anatomic bypass or primary repair. However, open surgical repair is often difficult, because the patients have a history of pelvic intervention and hemodynamic instability due to hemorrhage (Fox et al. 2011). Therefore, an endovascular approach using covered stents and coil embolization has become the treatment of choice for UAF (Van den Bergh et al. 2009). Previous studies have reported that the immediate success rate of EVT using stent-graft or metallic stents was 85%–100% (Fox et al. 2011; Okada et al. 2013). However, the hematuria recurrence-free rates at 1 and 2 years were 76.2% and 40.6%, respectively (Okada et al. 2013). The mechanism of this high recurrence rate appears to be the ongoing process of inflammation and advancement of malignancy. The stent grafts are more preferable to prevent re-bleeding than metallic stents; however, have inherent risk of recurrent infection (Fox et al. 2011). In this case, IVUS revealed that the ureter had the communication to the retrograde subintimal space of the let EIA; therefore, the stent graft did not touch the ureter and the ureteral stent directly. We considered that the stent graft might not be affected by the post-operative urinary infection. Moreover, the present case report suggests that the re-bleeding could be owing to the late enlargement of the subintimal space of iliac arteries caused by the friction injury of the ureteral stent. Because angiography and IVUS detected the entry of the subintimal space in the EIA 25.0 mm distal from the crossing of the ureter, a covered stent with a length of 50.0 mm was implanted to seal both the crossing and the distal subintimal entry. In addition, because the subintimal lumen could advance into the ipsilateral IIA in the future, coil embolization of the IIA was performed before stent-graft implantation.

Another issue of the EVT is re-occlusion of iliac arteries; however, the previous report demonstrated that limb ischemia was more common with surgical repair (67%) than the EVT (50%) in cases with UAF (Fox et al. 2011). Moreover, the primary patency after stent-graft implantation was superior to that after metallic stents in iliac arteries (Bracale et al. 2019). Because the dual-antiplatelet therapy at least 6 months could provide the high primary patency rate of stent grafts in iliac arteries (Bracale et al. 2019), we considered that the EVT using Viabahn covered stent™ might be the appropriate treatment in the present case.

## Conclusions

We demonstrated a case of UAF, in which IVUS allowed to visualize the communication between the ureter and the subintimal lumen in the EIA. The fistula could be treated using a covered stent and coil embolization; however, careful follow-up is necessary because the subintimal lumen may be enlarged by the stress of ureteral stents.

## Additional files

Additional file 1: **Movie S1.** Axial imaging of the computed tomography. (MOV 2372 kb)

Additional file 2: **Movie S2.** Sagittal imaging of the computed tomography. (MOV 4153 kb)

Additional file 3: **Movie S3.** Baseline angiography. (MPG 3614 kb)

Additional file 4: **Movie S4.** Intravascular ultrasound imaging from the left ureter to the subinrimal lumen of the external iliac artery. (MOV 4662 kb)

## Abbreviations

CIA: Common iliac artery
CT: Computed tomography
EIA: External iliac artery
EVT: Endovascular therapy
IIA: Internal iliac artery
IVUS: Intravascular ultrasound
UAF: Ureteroarterial fistula
